# Clinical and prognostic significance of perioperative change in red cell distribution width in patients with esophageal squamous cell carcinoma

**DOI:** 10.1186/s12885-023-10804-7

**Published:** 2023-04-06

**Authors:** Peng Zhang, Sheng Wang, Jun-zhou Wu, Qian Song

**Affiliations:** 1 Department of Radiology Physics, Key Laboratory of Radiation Oncology of Zhejiang Province, Zhejiang Cancer Hospital, Institute of Basic Medicine and Cancer (IBMC), Chinese Academy of Sciences, Hangzhou, Zhejiang China; 2 Department of Clinical Laboratory, Zhejiang Cancer Hospital, Institute of Basic Medicine and Cancer (IBMC), Chinese Academy of Sciences, Hangzhou, Zhejiang China; 3 Cancer Research Institute, Zhejiang Cancer Hospital, Institute of Basic Medicine and Cancer (IBMC), Chinese Academy of Sciences, Hangzhou, Zhejiang China

**Keywords:** Red cell distribution width, Esophageal squamous cell carcinoma, Perioperative change, Survival, Prognosis

## Abstract

**Background:**

Numerous studies have reported the prognostic significance of the red cell distribution width (RDW) in patients with esophageal squamous cell carcinoma (ESCC), but the relationship between the perioperative change in RDW (delta RDW) and survival in patients with ESCC after surgery has not been evaluated.

**Methods:**

A total of 594 patients with newly diagnosed ESCC after surgery were enrolled in the study. Delta RDW (delta RDW = Postoperative RDW–Preoperative RDW) was counted based on data within one week before surgery and two weeks after surgery. To investigate the relationship between delta RDW and overall survival (OS), the median delta RDW was chosen as the cut-off value.

**Results:**

99 (16.7%) patients had pathological stage 1a-1b, 202 (34.0%) patients had pathological stage 2a-2b, and 293 (49.3%) patients had pathological stage 3a-3c.There were 179 (30.1%) patients who had vessel invasive, and 415 (69.9%) patients without vessel invasive. There were 216 (36.4%) patients with nerve infiltration, and 378 (63.6%) without nerve infiltration. In univariate analysis, five parameters including delta RDW(≥ 0.44 vs.<0.44) (P = 0.039, HR = 1.337, 95% CI = 1.014–1.762) significantly correlated with worse OS. Multivariate analysis revealed that delta RDW(≥ 0.44 vs.<0.44) was an independent prognostic marker for OS (P = 0.033, HR = 1.356, 95% CI = 1.025–1.793). Kaplan-Meier curves showed that delta RDW ≥ 0.44 was significantly associated with worse OS (P = 0.039). Subgroup analysis suggested that delta RDW ≥ 0.44 indicated worse survival in patients with ESCC exclusively in these subtypes such as female patients, age > 60 patients, patients with lymph node metastasis, and patients with vessel invasive.

**Conclusions:**

Perioperative change in red cell distribution width predicts worse survival in patients with ESCC after surgery.

## Introduction

Esophageal cancer ranks seventh in terms of incidence and sixth in terms of mortality, with approximately 604,000 new cases and 544,000 deaths expected worldwide in 2020 [1]. In addition, esophageal cancer ranks fourth in terms of cancer-related mortality in China [2]. The predominant pathological type of esophageal cancer is squamous cell carcinoma in China [3, 4]. The prognosis of patients with ESCC remains unsatisfactory due to high rates of recurrence and distant metastasis. Although the incidence rate of ESCC has decreased in certain high-risk areas in China and treatments including surgery, radiotherapy, chemotherapy and immunotherapy have improved, the survival rate of patients with ESCC at 5 years after diagnosis is only 20–30% [5–9]. Therefore, reliable and routine prognostic indicators to guide the perioperative management and screening of patients at high risk of death are urgently needed.

Red cell distribution width (RDW), which is one of the red blood cell (RBC) indices, reflects RBC volume heterogeneity. Differences in RDW correlate RBC survival patterns and indicate derailment of erythropoiesis [10]. Accumulating evidence indicates that RDW is high in patients with active inflammation and is associated with hypertension, cardiometabolic dysfunction and cancer [11–17]. Many studies have shown that RDW is associated with the release of inflammatory markers including TNF-α, IL-6, IL-10, CD8 + T cells [18, 19]. In addition, an elevated RDW is indicative of a poor nutritional status, which deteriorates as the tumor progresses. In addition, previous studies have reported that an increased RDW is correlated with worse survival in patients with lung cancer, colon cancer, and esophageal cancer [15, 16, 20]. These reports have focused on preoperative RDW for its application in the area of cancer, while the correlation between the perioperative change in RDW value (delta RDW) and prognosis in patients with ESCC has not been investigated. Our research is the first to show that delta RDW predicts worse survival in patients with ESCC.

We speculated that delta RDW may predict the effect of surgical treatment and indicate overall survival in patients with ESCC. Therefore, we evaluated whether delta RDW could serve as an independent prognostic indicator in patients with ESCC.

## Materials and methods

### Patient selection

594 patients with newly diagnosed ESCC were included in our collection at Zhejiang Cancer Hospital between 2008 and 2014. All tumor tissues were pathologically confirmed after surgery. The preoperative blood routine was checked within one week before surgery. The preoperative RDW data, which is the closest to the date of surgery, was collected in the present study. The postoperative blood routine was evaluated within fourteen days of surgery. Because of the influence of the stress response after surgery, the postoperative RDW closest to the time of discharge was recorded. The RDW data is the calculated value and the unit of RDW is %. The exclusion criteria were as follows: first, patients without complete clinical factors and laboratory data. Second, patients had active infection or other types of cancer or any coexisting hematological disease that could influence the RDW value. Third, patients had undergone the neoadjuvant chemotherapy and neoadjuvant chemoradiotherapy before surgery. ESCC patients staged with AJCC 7th staging system. Our study was authorized by the Ethics Committee of Zhejiang Cancer Hospital. All patients who meet the inclusion criteria obtained informed consent.

### Statistical analysis

Preoperative RDW and postoperative RDW that did not meet the normal distribution standard were presented by median and the interquartile range. The patient clinical characteristics that belong to categorical variables were shown as numbers and percentages. We used the chi-square test to analyse categorical numbers. Overall survival (OS) was counted from the date of surgery to the date of death and last follow-up. The Kaplan-Meier method and the log-rank test were utilized to investigate OS. We plotted the survival curve using the GraphPad Prism 7 software. The prognostic value was assessed by COX regression analysis. P less than 0.05 reach to statistical significance. Statistical analysis was performed using SPSS, version 19.0 (SPSS, Chicago, IL, USA).

## Results

### Patient clinical features

A total of 513 (86.4%) male and 81 (13.6%) female patients who were newly diagnosed with ESCC were enrolled in the present study. There were 270 (45.5%) young patients (≤ 60 years) and 324 (54.5%) old patients whose age at first diagnosis was more than 60 years. There were 44 (7.4%) patients with well differentiated pathology grade, 403 (67.8%) patients with intermediate differentiated pathology grade, 145(24.5%) patients with poorly differentiated pathology grade, and 2 (0.3%) patients with undifferentiated pathology grade. 257 (43.3%) patients without lymph node metastasis, and 337 (56.7%) patients with lymph node metastasis. There were 99 (16.7%) patients with pathological stage 1a-1b, 202 (34.0%) patients with pathological stage 2a-2b, and 293 (49.3%) patients with pathological stage 3a-3c. 179 (30.1%) patients with vessel invasive, and 415 (69.9%) patients without vessel invasive. There were 87 (14.6%) patients with perioperative complications, and 507 (85.4%) patients without perioperative complications. The interquartile range of the preoperative RDW was 12.3–13.3, and the median value was 12.8. The interquartile range of the postoperative RDW was 12.7–13.8, and the median value was 13.2. Details of patient characteristics are shown in Table 1.

**Table 1:** Demographic and clinical data of 594 ESCC patients accroding to delta RDW

Charateristics		Total (N = 594), %	delta RDW		*P* value
			< 0.44 (N = 363), %	≥ 0.44 (N = 231), %	
Sex	Male	513 (86.4)	324 (89.3)	189 (81.8)	**0.010**
	Female	81 (13.6)	39 (10.7)	42 (18.2)	
Age (years)	≤ 60	270 (45.5)	177 (48.8)	93 (40.3)	**0.043**
	> 60	324 (54.5)	186 (51.2)	138 (59.7)	
Pathology grade	Well	44 (7.4)	26 (7.2)	18 (7.8)	0.972
	middle	403 (67.8)	246 (67.8)	157 (68.0)	
	Poorly	145 (24.5)	90 (24.7)	55 (23.8)	
	Undifferentiated	2 (0.3)	1 (0.3)	1 (0.4)	
Depth of tumor	T1a–1b	55 (9.3)	37 (10.2)	18 (7.8)	0.420
	T2	114 (19.2)	73 (20.1)	41 (17.7)	
	T3	425 (71.5)	253 (69.7)	172 (74.5)	
Lymph node	N0	257 (43.3)	154 (42.4)	103 (44.6)	0.922
	N1	190 (32.0)	118 (32.5)	72 (31.2)	
	N2	99 (16.7)	60 (16.5)	39 (16.9)	
	N3	48 (8.1)	31 (8.5)	17 (7.4)	
Pathological stage	1a–1b	99 (16.7)	60 (16.5)	39 (16.9)	0.815
	2a–2b	202 (34.0)	127 (35.0)	75 (32.5)	
	3a–3c	293 (49.3)	176 (48.5)	117 (50.6)	
Vessel invasive	Yes	179 (30.1)	118 (32.5)	61 (26.4)	0.114
	No	415 (69.9)	245 (67.5)	170 (73.6)	
Nerve infiltration	Yes	216 (36.4)	127 (35.0)	89 (38.5)	0.382
	No	378 (63.6)	236 (65.0)	142 (61.5)	
Complications	Yes	87 (14.6)	30 (8.3)	57 (24.7)	**< 0.001**
	No	507 (85.4)	333 (91.7)	174 (75.3)	
Treatment regimen	S	402 (67.7)	236 (65.0)	166 (71.9)	0.163
	S + postoperative C	137 (23.1)	93 (25.6)	44 (19.0)	
	S + postoperative CRT	55 (9.3)	34 (9.4)	21 (9.1)	
Preoperative RDW	Median	12.8 (12.3–13.3)	12.9 (12.4–13.4)	12.7 (12.3–13.2)	**0.010**
Postoperative RDW	Median	13.2 (12.7–13.8)	12.9 (12.5–13.4)	13.7 (13.2–14.3)	**< 0.001**
Preoperative NLR	Median	2.17 (1.60–2.89)	2.17 (1.58–2.92)	2.15 (1.63–2.88)	0.378
Postoperative NLR	Median	5.55 (4.00-7.17)	5.67 (4.28–7.55)	5.10 (3.63–6.77)	**0.022**
Preoperative PNI	Median	50.1 (47.0-53.5)	49.9 (46.9–53.5)	50.7 (47.2–53.5)	0.107
Postoperative PNI	Median	40.4 (37.0-43.6)	39.7 (36.6–43.3)	40.9 (37.5–44.7)	**0.002**

Abbreviations: S, surgery; C, chemotherapy; CRT, chemoradiotherapy

### Correlation between delta RDW and patient characteristics

The clinical characteristics of ESCC patients in the delta RDW < 0.44 group and in the delta RDW ≥ 0.44 group are shown in Table 1. There were obvious differences in gender (P = 0.010), age (P = 0.043), and complications (P < 0.043) between the delta RDW < 0.44 group and the delta RDW ≥ 0.44 group. Delta RDW ≥ 0.44 group had more perioperative complications than those with delta RDW < 0.44 group. No significant difference was observed between the two groups in terms of patient characteristics such as depth of tumor, pathology grade, pathological stage, lymph node metastasis, nerve infiltration, vessel invasive, and treatment regimen. The median preoperative RDW was lower in the delta RDW ≥ 0.44 group compared to the delta RDW < 0.44 group (P = 0.010). Nevertheless, the median postoperative RDW was higher in the delta RDW ≥ 0.44 group than in the delta RDW < 0.44 group (P < 0.001).

### Difference in survival according to delta RDW

**Fig. 1:**
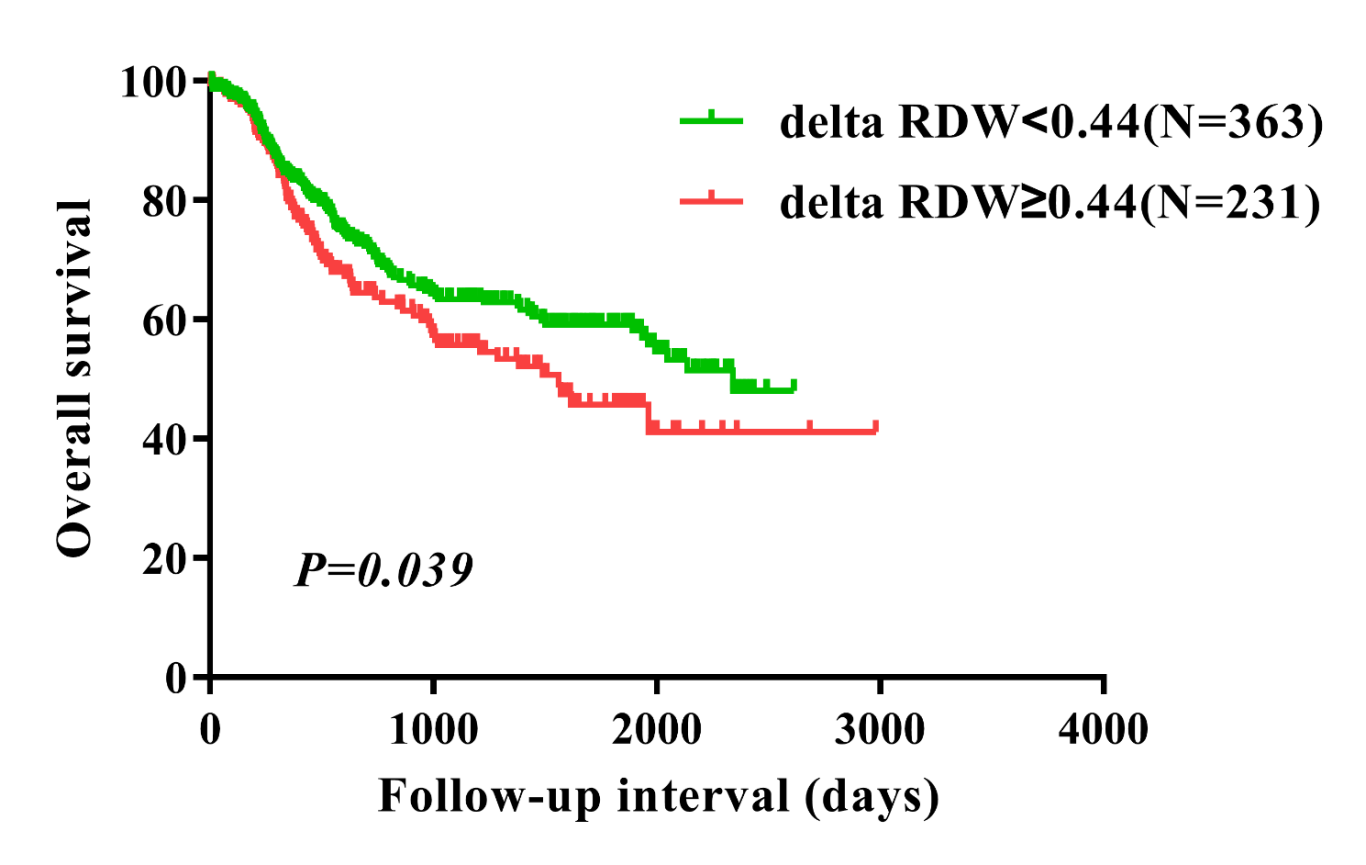
Overall survival analysis in all 594 patients with ESCC according to delta RDW. ESCC, esophageal squamous cell carcinoma. RDW, red blood cell distribution width

Patients in the delta RDW ≥ 0.44 group had a significantly worse OS compared to patients in the delta RDW < 0.44 group (P = 0.039) (Fig. 1). Univariate analysis revealed that five clinical factors, including delta RDW (≥ 0.44 vs.<0.44) (P = 0.039), lymph node metastasis (P < 0.001), depth of tumor (P < 0.05), pathological stage (P < 0.001), nerve infiltration (absence vs. presence) (P < 0.001), and vessel invasive (absence vs. presence) (P < 0.001) were associated with worse survival. Multivariate analysis indicated that delta RDW (≥ 0.44 vs.<0.44) (P = 0.033), nerve infiltration (absence vs. presence) (P = 0.007), and lymph node metastasis (P < 0.001) could independently predict clinical outcome in ESCC patients (Table 2). There were positive correlations between delta RDW and postoperative-LNR, delta-LNR, delta-NLR, postoperative-LMR and postoperative-PNI. Postoperative-NLR was negatively correlated with delta RDW (Table 3).

**Table 2:** Overall survival analyses according to delta RDW in 594 patients with ESCC

Variables	Univariate			Multivariate		
	HR	95% CI	*P* value	HR	95% CI	*P* value
delta RDW(≥ 0.44 vs.<0.44)	1.337	1.014–1.762	**0.039**	1.356	1.025–1.793	**0.033**
Sex (male vs.female)	1.204	0.793–1.829	0.383			
Age (> 60 vs.≤60)	1.157	0.880–1.520	0.297			
**Pathology grade**						
Well differentiated	0.154	0.019–1.225	0.077			
middle differentiated	0.260	0.036–1.871	0.181			
Poorly differentiated	0.384	0.053–2.779	0.343			
Undifferentiated	1.000					
**Depth of tumor**						
T1a–1b	0.486	0.257–0.920	**0.027**	0.524	0.225–1.217	0.133
T2	0.593	0.404–0.869	**0.007**	0.567	0.292–1.099	0.093
T3	1.000					
**Lymph node metastasis**						
N0	0.134	0.087–0.204	**< 0.001**	0.099	0.034–0.287	**< 0.001**
N1	0.250	0.167–0.375	**< 0.001**	0.263	0.171–0.405	**< 0.001**
N2	0.414	0.269–0.635	**< 0.001**	0.448	0.291–0.689	**< 0.001**
N3	1.000					
**Pathological stage**						
1a–1b	0.310	0.190–0.507	**< 0.001**	2.632	0.573–12.092	0.213
2a–2b	0.386	0.278–0.536	**< 0.001**	1.468	0.602–3.581	0.399
3a–3c	1.000					
Vessel invasive (absence vs. presence)	1.808	1.369–2.388	**< 0.001**	1.194	0.884–1.612	0.248
Nerve infiltration (absence vs. presence)	1.899	1.447–2.493	**< 0.001**	1.483	1.112–1.977	**0.007**
**Treatment regimen**						
S	1.131	0.692–1.848	0.623			
S + postoperative C	1.327	0.777–2.269	0.300			
S + postoperative CRT	1.000					
delta NLR	1.016	0.973–1.061	0.473			
delta PNI	1.008	0.986–1.030	0.499			

Abbreviations: S, surgery; C, chemotherapy; CRT, chemoradiotherapy

**Table 3:** The correlation between delta RDW and some inflammatory associated markers and nutrition markers

Variables	delta RDW	
	spearman correlation	P-value
Pre-LNR	-0.044	0.282
Post-LNR	0.133	**0.001**
Delta-LNR	0.115	**0.005**
Pre-NLR	0.044	0.284
Post-NLR	-0.133	**0.001**
Delta-NLR	0.137	**0.001**
Pre-LMR	-0.012	0.777
Post-LMR	0.164	**< 0.001**
Delta-LMR	-0.074	0.073
Pre-PLR	-0.012	0.768
Post-PLR	0.041	0.324
Delta-PLR	-0.062	0.13
Pre-Albumin	0.025	0.539
Post-Albumin	0.07	0.087
Delta-Albumin	0.043	0.291
Pre-PNI	0.022	0.592
Post-PNI	0.099	**0.016**
Delta-PNI	0.074	0.072

### Subgroup analysis based on other clinical factors

**Fig. 2:**
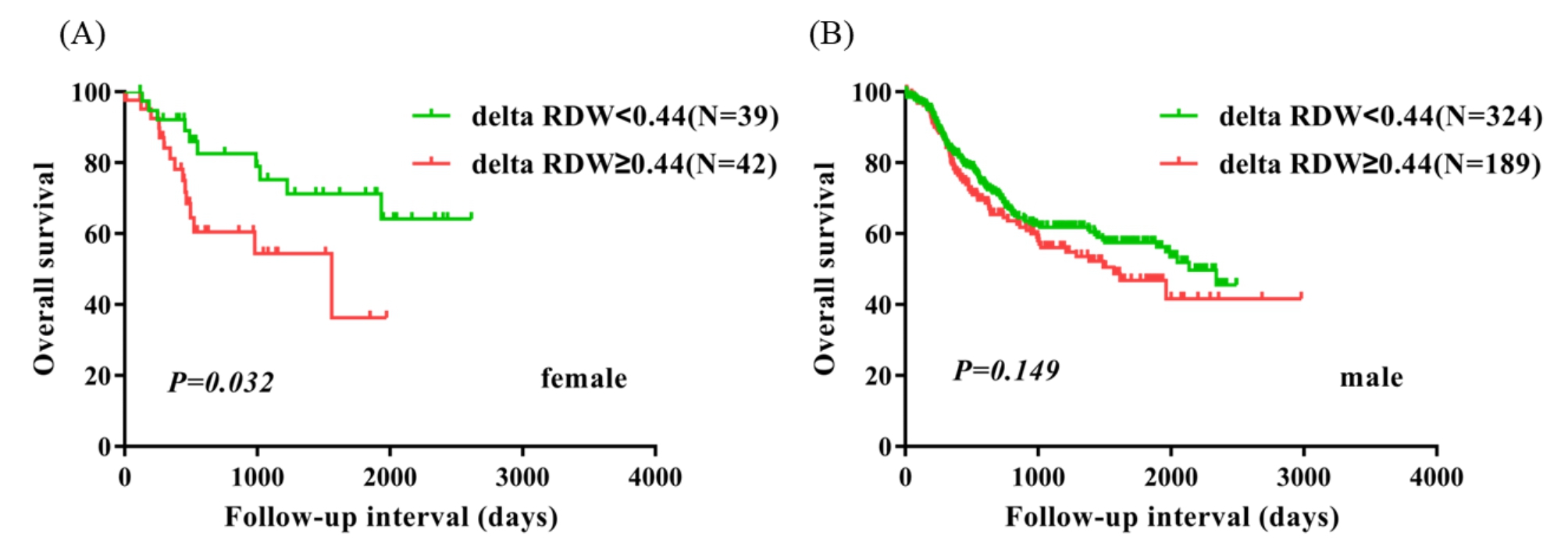
Overall survival analysis in female patients and male patients according to delta RDW (A, B)

**Fig. 3:**
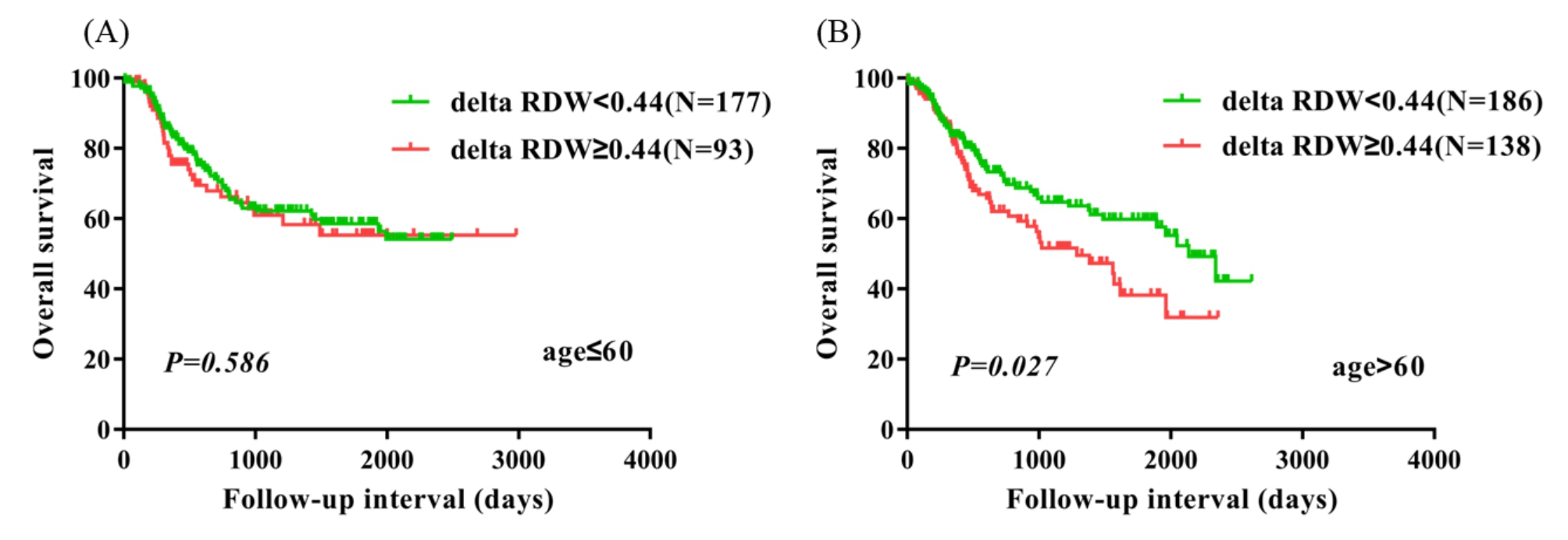
Overall survival analysis in age ≤ 60 patients and age > 60 patients according to delta RDW (A, B)

**Fig. 4:**
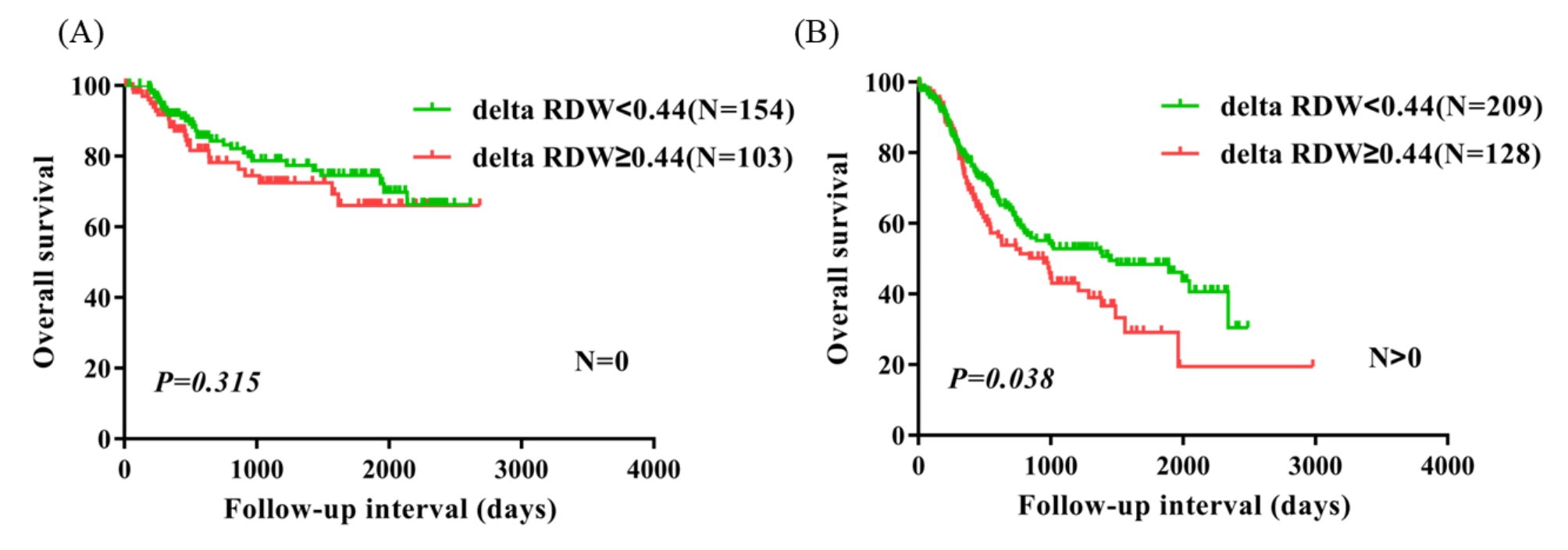
Overall survival analysis in patients without lymph node metastasis, patients with lymph node metastasis according to delta RDW (A, B)

**Fig. 5:**
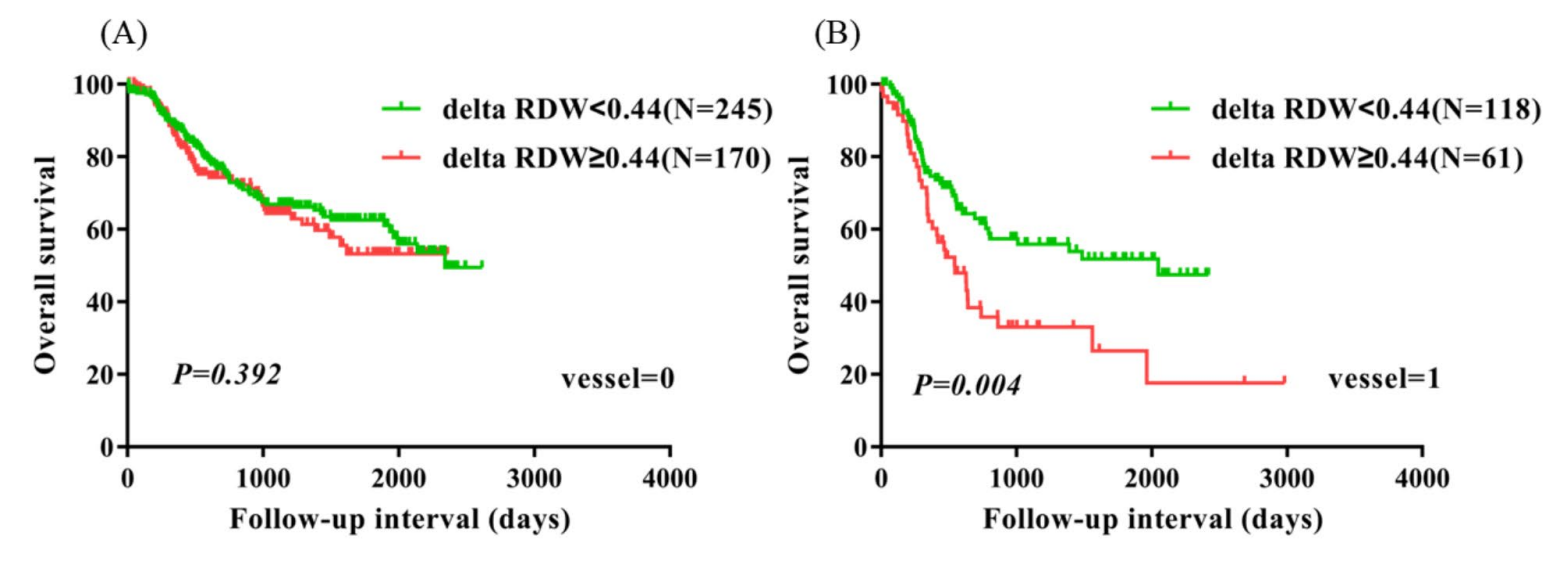
Overall survival analysis in patients without vessel invasive and patients with vessel invasive according to delta RDW (A, B)

To identify the subtypes of patients affected by delta RDW, patients were divided according to sex, age, lymph node metastasis, pathological stage, vessel invasive, and nerve infiltration. Female patients, age > 60 patients, patients with lymph node metastasis, patients with vessel invasive had significantly worse survival in the delta RDW ≥ 0.44 group compared with the delta RDW < 0.44 group (P = 0.032, P = 0.027, P = 0.038, and P = 0.004). Nevertheless, male patients, age ≤ 60 years patients, patients without vessel invasive, and patients without nerve infiltration were not significantly different between the two groups (Figs. 2, 3, 4 and 5). No obvious difference was observed in all subgroups of pathological stage and nerve infiltration (data not shown).

## Discussion

In the present study, we first demonstrated that delta RDW ≥ 0.44 was significantly correlated with poor prognosis in ESCC patients. Delta RDW ≥ 0.44 in cancer patients may be caused by increased inflammation induced by the tumor cells themselves, the tumor microenvironment and surgery. Increased inflammation depresses the response to erythropoietin, attenuates iron release, and decreases the survival time of red blood cells by relevant inflammatory markers, leading to a higher delta RDW value [21]. Therefore, a higher delta RDW may indicate increased postoperative inflammation in ESCC patients. We analyzed the correlation between delta RDW change with blood inflammation markers such as LNR, NLR, LMR, and PLR or nutrition markers such as albumin and PNI. There were positive correlations between delta RDW and postoperative-LNR, delta-LNR, delta-NLR, postoperative-LMR, and postoperative-PNI. Postoperative-NLR was negatively correlated with delta RDW (Table 3). It is generally accepted that inflammation plays a vital value in tumorigenesis and the tumor microenvironment [22–24]. In addition, accumulating studies suggest that inflammation is closely correlated with prognosis in patients with cancer, including those with ESCC [25]. Peripheral lymphocytes, neutrophils, and monocytes indicate the inflammatory status and serve as an independent prognostic factor in various cancers [26–29]. Some calculated values such as NLR, LMR, and PLR are significantly correlated with the survival of cancer patients [30–32]. Many studies reported that RDW could predict the prognosis of cancer patients [15, 16, 20]. However, these studies were based on the preoperative RDW. The correlation between delta RDW and survival in ESCC patients has not been evaluated. Our research is the first to show that the perioperative change in red cell distribution width (delta RDW) predicts worse survival in patients with ESCC.

We have reported here that delta RDW value may also be a predictor of inflammation that is closely correlated with survival in ESCC patients. Our previous reports demonstrated that delta LMR value and delta neutrophil value were also closely correlated with the prognosis of ESCC patients [33, 34]. Postoperative infectious complications (ICs) have been shown to worsen the survival of esophageal cancer patients [35]. These results clearly indicate that changes in inflammatory markers during the perioperative period affect the survival of cancer patients. Therefore, the close relationship between delta RDW value and survival shown in the present study may be due to the influence of postoperative inflammation caused by surgery and postoperative infectious complications.

The common symptoms of luminal obstruction and dysphagia can lead to malnutrition in patients with ESCC. Examination of nutritional status contributes to the prediction of survival in patients with ESCC. Preoperative prealbumin concentration may serve as an independent prognostic indicator in ESCC patients [36]. Preoperative PNI, a calculated value, was useful in indicating survival in ESCC patients [37]. Preoperative RDW has been shown to have prognostic value in patients with ESCC [20]. RDW has been reported to correlate with nutritional status [38]. Therefore, the close relationship between preoperative RDW and survival may be caused by the influence of nutritional status on survival. In addition, surgery for ESCC patients sometimes leads to malnutrition because patients do not recover after surgery, which may worsen the survival of ESCC patients. In this regard, a previous study showed that postoperative PNI has a significant close correlation with survival in patients with ESCC [39]. Therefore, postoperative RDW could be correlated with survival in patients with ESCC. Preoperative RDW and postoperative RDW were both correlated with survival in ESCC patients. Therefore, delta RDW, calculated as postoperative RDW minus preoperative RDW, was used to investigate the prognostic value. In the present study, we demonstrated that delta RDW was an independent prognostic indicator in ESCC patients. Furthermore, the close relationship between delta RDW and survival in this study was probably due to postoperative malnutrition. Some studies have reported that RDW was closely correlated with survival in patients with ESCC. These findings focused on preoperative RDW, whereas in this study we evaluated the prognostic value of delta RDW by combining both preoperative RDW and postoperative RDW. To the best of our knowledge, this study is the first to report that delta RDW during the perioperative period may be an independent prognostic indicator in patients with ESCC.

Several shortcomings of our study need to be acknowledged. First, we did not design a validation set to support the certification of the prognostic value of delta RDW. Second, due to the retrospective design of the study, the blood routine was not examined at a specific time. To reduce the risk of a postoperative stress response, the postoperative RDW value farthest from the date of surgery was used. We hope that in the future study we will be able to check the RDW values during routine follow-up on specific days, for example during 14 days. More prospective and multicenter studies are needed to validate the correlation between delta RDW and prognosis. Third, because of the small number of patients with adjuvant therapies such as radiotherapy and/or chemotherapy, the prognostic value of different adjuvant therapies was not evaluated based on subgroups. Despite these shortcomings, we first evaluated the correlation between delta RDW and survival in patients with ESCC. In addition, the determination of RDW is included in the blood routine, which is cheap, routine and reliable in clinical examination. The clinical application of delta RDW could guide the evaluation of survival in ESCC patients, especially in these subgroups such as female patients, patients aged > 60 years, patients with lymph node metastasis, and patients with vessel invasive.

## Conclusion

Taken together, this study suggests that delta RDW ≥ 0.44 indicates worse survival in patients with ESCC exclusively in these subtypes such as female patients, age > 60 patients, patients with lymph node metastasis, and patients with vessel invasive. Delta RDW ≥ 0.44 contributes to evaluate the patient risk stratification, design an effective therapy option, and indicate overall survival based on the clinical laboratory data.

## Data Availability

All data generated or analyzed in the present study are available from the corresponding author upon reasonable request.
